# EXPLORING STRESSORS AND SELF-MANAGEMENT BEHAVIORS IN RURAL AGING AFRICAN AMERICANS WITH DIABETES: A PILOT STUDY

**DOI:** 10.1093/geroni/igae098.3011

**Published:** 2024-12-31

**Authors:** Idethia Shevon Harvey, Sophia Nittinger, Maude Harris, Arika Wiggins, Rahma Mkuu

**Affiliations:** University of Missouri, Columbia, Missouri, United States; University of Missouri, Columbia, Missouri, United States; University of Missouri, Columbia, Missouri, United States; Southeast Missouri State University, Cape Girardeau, Missouri, United States; University of Florida, Gainesville, Florida, United States

## Abstract

**Background:**

Rural African Americans with diabetes encounter unique challenges related to managing their condition, including diabetes-specific distress, financial stress, and interpersonal stressors. This study investigated the associations between these stressors and self-management behaviors in a small population sample.

**Methods:**

Fifteen rural African American adults (mean age = 66.08 yrs.; SD = + 6.16) with diabetes participated in this cross-sectional study. Participants completed validated measures, including the Summary of Diabetes Self-Care Activity (SDSCA), Center for Epidemiologic Studies Depression Scale (CES-D), Perceived Stress Scale (PSS), Partner Strain, Parental Stress Scale, Diabetes Distress, Everyday Discrimination, and Cost-FACIT. Descriptive statistics and correlational analyses examined the relationships between stressors and self-management behaviors.

**Results:**

Participants reported moderate levels of financial, relational, and perceived stress, and diabetes distress. Higher levels of financial stress were associated with poorer diabetes self-care activities (r = -0.628, p < 0.05). Emotional burden (r = 0.804, p < 0.01) and interpersonal distress (r = 0.600, p < 0.05) are significantly correlated with perceived stress. Financial stress is significantly associated with everyday discrimination (r = 0.508, p < 0.05).

**Conclusion:**

Despite the small sample size, our findings highlight the intricate interplay between diabetes distress, financial stress, relationship stress, and self-management behaviors among rural African Americans with diabetes. Tailored interventions addressing these stressors and promoting effective coping strategies may be crucial in improving diabetes management outcomes in this population. Further research with larger samples is warranted to confirm these findings and inform targeted interventions for better diabetes care in rural African American communities.

